# Incidence and factors associated with geographical relocation in patients receiving renal replacement therapy

**DOI:** 10.1186/s12882-020-01887-6

**Published:** 2020-07-01

**Authors:** Hicham I Cheikh Hassan, Jenny HC Chen, Karumathil Murali

**Affiliations:** 1 Renal Unit, Wollongong Hospital, Locked Bag 8808, South Coast Mail Centre, Wollongong, NSW 2521 Australia; 2 University of Wollongong, Wollongong, NSW Australia; 3 The George Institute for Global Health, Wollongong, Australia

**Keywords:** End stage kidney disease, Renal replacement therapy, Geographic relocation, Patient impact, Healthcare access

## Abstract

**Background:**

Renal replacement therapy (RRT) places a burden on patients, and geographical relocation for easier access to healthcare facilities is a necessity for some. Incidence and factors associated with relocation has not been comprehensively examined at a national level. We aimed to determine proportion, incidence, characteristics of RRT patients who relocate and relocation rate by remoteness of residence and dialysis modality.

**Methods:**

Retrospective cohort analysis using Australian and New Zealand Dialysis and Transplant Registry to examine RRT patients in Australia from January 2005 to December 2015. Relocation incidence was calculated for remoteness of residence and RRT modality as rate per 100 patient years. Factors associated with relocation were examined using competing risk regression models with death as a competing event.

**Results:**

Of 24,676 incident patients on RRT, 5888 (23.9%) relocated with a median time of 1.6 years [IQR 0.7–3.4] years. Relocation incidence was 7.9 per 100 patient years and increased from major cities to very remote regions (7.2 to 48.8 per 100 patient years respectively, *p* < 0.001). Remoteness of residence was associated with geographical relocation in competing risk analysis especially in remote (SHR 1.20, 95%CI 1.01, 1.41 *p* = 0.034) and very remote regions (SHR 3.51 95% 3.05, 4.04 *p* < 0.001). Aboriginal or Torres Strait Islander ethnicity, compared to Caucasian, was independently associated with relocation (SHR 1.18, 95% CI 1.06,1.31, *p* = 0.002) while transplant patients were less likely to relocate compared to haemodialysis patients (HR 0.37, 95%CI 0.34, 0.39, *p* < 0.001).

**Conclusions:**

Relocation in patients receiving RRT is associated with remoteness of residence, RRT modality and ethnicity. Reasons for relocation and its impact on patient wellbeing and outcome should be further explored.

## Background

Globally, the number of people requiring renal replacement therapy (RRT) is rising [1]. RRT places a high burden on health resources in the form of regular clinic appointments, investigations, interventions and routine dialysis attendance in specialised centres. Given the complexity of RRT treatment many patients find themselves facing factors necessitating a need to geographically relocate.

Relocation in patients receiving RRT has not been studied extensively. Observational studies have shown that increased travel time to a treatment center for haemodialysis (HD) is associated with lower HD prevalence, poorer quality of life and higher all-cause mortality [2–6]. Another factor associated with relocation is access to treatment by remoteness of residence. In Australia, rural and regional residents with chronic diseases had fewer physician visits, more difficulty accessing health services and a higher mortality compared to their urban counterparts [7–10]. RRT patients who lived in regional districts, compared to patients in urban centers, used a lower proportion of dialysis care and had worse survival [4, 5, 11, 12]. Both the travel time and access to healthcare are potential reasons for the lower prevalence of dialysis in rural Australia compared to major cities [4, 12].

While relocation offers health benefits and easier access to health services, it also causes significant stress on the patient and family. Patients who relocated from rural areas for better health care access often face separation from family, lack of social support and financial burdens [13, 14]. Dialysis patients who relocated described a decline in willingness to maintain treatment regimens [15]. The final decision to relocate is likely to involve a trade-off between benefits of being closer to family, friends and a familiar social environment (favouring non-relocation) and health benefits of being closer to a treatment centre in a major health centre (favouring relocation).

The aims of our study were three-fold. Firstly, we examined characteristics of patients with end-stage kidney disease who relocated and who did not relocate after commencement of RRT. Secondly, we assessed the incidence and pattern of relocation between metropolitan, rural and remote areas. Lastly, we determined the association between geographical remoteness and RRT modality with relocation rates.

## Methods

### Study design and setting

We conducted a retrospective cohort study using the Australia and New Zealand Dialysis and Transplant Registry (ANZDATA). ANZDATA collects yearly information (ending December 31) on all receiving RRT in Australia and New Zealand. Notification of RRT modality change, treatment centre change and death occurs in real time. Permission to analyse the data was granted by the ANZDATA executive. Only de-identified data were analysed, and the need for informed consent was waived.

### Participants

Adult (≥18 years) patients who initiated RRT in Australia between January 12,005 and December 31,2015, were alive or remained on RRT for > 6 months and who did not recover renal function were included in the study. The year 2005 was chosen since postcode information was recorded from 2005 onwards on a yearly basis at the end of the calendar year. We excluded New Zealand patients (due to incomplete postcode information), patients with no postcode history, those with > 6 months between start date and postcode documentation date and patients who relocated to or from an overseas location.

### Data collection

Baseline patient characteristics were collected at the initiation of RRT and included age, gender, ethnicity, comorbidity and cause of renal disease. Comorbidities recorded include: diabetes, peripheral vascular disease, chronic lung disease, coronary artery disease and cerebrovascular disease. Ethnicity was classified as Caucasian, Asian, Aboriginal and Torres Strait Islander (ATSI), Maori-Pacific Islander (MPI) and Other. Causes of renal disease were classified as glomerulonephritis, diabetes, hypertension, cystic disease and other.

RRT modality was categorised as transplant, home therapies and facility HD at the start of initiating RRT and at the end of the study period (first relocation or censoring). We combined peritoneal dialysis and home HD since both belong to the same the category of home-based therapies and have similar implications for relocation. If treatment centre or state changed in the year of relocation, then the RRT modality at the time of change was chosen. If RRT modality changed during the year of relocation, then the newer RRT modality was chosen if RRT modality changed in the first 6 months of the year and the older RRT modality if the change occurred in the second 6 months of the year. For patients who did not relocate we assigned the RRT modality at censoring.

Remoteness of residence was determined from the postcode using the Accessibility/ Remoteness Index of Australia taken from the Australia Bureau of Statistics using Australian Standard Geographical Classification from the 2011 census data [16]. There are five classes of major city, inner regional, outer regional, remote and very remote based on physical road distance from a location to the nearest urban centre. We also further examined relocations from and to major city by change of state, which would indicate that a relocation occurred to a new city compared to within the same city.

### Exposure factor

Geographical relocation was defined as the first change in postcode. Postcode was captured by ANZDATA at the end of the calendar year (December 31st) while treatment centre or state change occurred in real time. Relocation date was set as end of the calendar year. If a treatment centre, state change or death occurred in the same year as a geographical relocation, then the exact date was used as the relocation date. Patients were censored on death, loss of follow-up or on reaching end of study period.

### Statistical analysis

Categorical data were expressed as number (percentage) and analysed using chi-square. Continuous data were was expressed as mean (standard deviation) or median (interquartile range) and analysed as per distribution. Incidence was calculated as the number of relocations divided by total patient years. We calculated the incidence for the total cohort, by remoteness of residence and by RRT modality as rate per 100 patient-years. Incidence of net patient gain or loss due to relocation for each remoteness of residence category was calculated from the difference between the total number of patients relocating to and leaving the remoteness of residence and dividing by the total patient years for the remoteness of residence. Factors associated with relocation were examined by fitting Cox proportional hazards models and competing risk analysis. A multivariable Cox proportional hazards model for time from RRT commencement to the first relocation was constructed using a backward selection procedure. Covariates with *p*-value ≤0.2 in the univariate analyses were included in the model. Interaction between remoteness of residence and ethnicity was tested using Wald test and found not to be significant (*p* = 0.7). Proportional hazards assumptions were confirmed graphically by plotting the Schoenfeld’s residuals. We also used Fine-Gray’s extension of the Cox proportional hazards model to fit competing risk regression models using death as a competing event. The covariates for Cox proportional hazards models and competing risk analysis were the same. Remoteness of residence and ethnicity were included in all models for their previously established associations. Results were expressed as hazard ratio (HR) and sub-hazard ratio (SHR) with 95% confidence interval (95%CI). We used listwise deletion to handle missing data. Statistical analysis was preformed using Stata (version 14.0).

## Results

Of the 30,188 adult patients who commenced RRT from 2005 to 2015, the following patients were excluded: 5160 from New Zealand, 87 with no postcode history, 19 who had no postcode history for > 6 months and 246 who relocated from or to overseas. In total 24,676 incident patients commenced RRT in Australia between 2005 and 2015, 5888 (23.9%) patients relocated and 18,788 (76.1%) did not. The patients were followed-up for a median [IQR] time of 2.2 [0.8,4.5] years with total follow-up time of 74,039.7 patient years. The study population was predominantly Caucasian (74.1%) with mean (SD) age of 60.3 (15.4) years and a high prevalence of comorbidities (65.8%) (Table 1). Most started RRT on facility HD (70.4%) and lived in a major city (66.6%).

**Table 1:** Baseline characteristics of study cohort with comparison between the relocation and non-relocation group

	All patients (*n* = 24,676)	Relocation (*n* = 5888,23.9%)	Non-relocation (*n* = 18,788, 76.1%)
Gender (Male)	15,172 (61.5%)	3498 (59.4%)	11,674 (62.1%)
Age (years)	60.3 (15.4)	55.4 (16.2)	61.8 (14.8)
Race			
Caucasian	18,142 (74.1%)	3876 (66.0%)	14,266 (76.6%)
Asian	2274 (9.3%)	517 (8.8%)	1757 (9.4%)
ATSI	2669 (10.9%)	1131 (19.3%)	1538 (8.3%)
MPI	765 (3.1%)	226 (3.8%)	539 (2.9%)
Other	635 (2.6%)	121 (2.1%)	514 (2.8%)
Comorbidities			
Any	16,228 (65.8%)	3781 (64.2%)	12,447 (66.2%)
Diabetes	11,570 (47.0%)	2853 (48.5%)	8717 (46.5%)
CLD	3021 (12.7%)	641 (11.3%)	2380 (13.1%)
CAD	8077 (34.7%)	1765 (31.6%)	6312 (35.7%)
PVD	4306 (18.6%)	993 (17.9%)	3313 (18.8%)
CerebVD	2775 (11.6%)	610 (10.6%)	5156 (11.9%)
Cause of Primary Renal Disease			
Glomerulonephritis	5494 (22.4%)	1446 (24.6%)	4048 (21.7%)
Diabetes	8803 (35.9%)	2265 (38.5%)	6538 (35.0%)
Hypertension	3262 (13.3%)	663 (11.3%)	2599 (13.9%)
Cystic disease	1751 (7.1%)	392 (6.7%)	1359 (7.3%)
Other	5242 (21.4%)	1117 (19.0%)	4125 (22.1%)
First RRT modality			
Facility HD	17,363 (70.4%)	5888 (73.8%)	13,015 (69.3%)
Home Therapies	6430 (26.1%)	1331 (22.6%)	5099 (27.1%)
Transplant	883 (3.6%)	209 (3.5%)	674 (3.6%)
RRT modality at relocation or censoring			
Facility HD	14,331 (58.1%)	3617 (61.4%)	10,714 (57.0%)
Home Therapies	5750 (23.3%)	1433 (24.3%)	4317 (23.0%)
Transplant	4595 (18.6%)	838 (14.2%)	3757 (20.0%)
Urbanity			
Major	16,433 (66.6%)	3652 (62.6%)	12,781 (68.1%)
Inner Regional	4301 (17.5%)	805 (13.8%)	3496 (18.6%)
Outer Regional	2397 (9.7%)	599 (10.3%)	1798 (9.6%)
Remote	635 (2.6%)	214 (3.7%)	421 (2.2%)
Very Remote	843 (3.4%)	567 (9.7%)	276 (1.5%)

Data expressed as numbers (percentage) or mean (standard deviation)

All variables statistically significant (*P* < 0.05) except for PVD

Number (%) of missing data: 191 (0.8%) in race, 55 (0.2%) in diabetes status, 875 (3.5%) in chronic lung disease, 1397 (5.7%) in coronary artery disease, 1533 (6.2%) in peripheral vascular disease, 685 (2.8%) in cerebral vascular disease and 124 (0.5%) in cause of primary renal disease,

*RRT* Renal replacement therapy, *HD* Hemodialysis, *ATSI* Australia and Torres Strait Islander, *MPI* Maori Pacific Islander, *CLD* Chronic Lung Disease, *CAD* Coronary artery disease, *PVD* Peripheral vascular disease, *CerebVD* Cerebrovascular disease

### Geographical relocation

Patients who relocated were more likely, when compared to those who did not relocate, to be younger (55.4 vs 61.8 years, *p* < 0.001), of ATSI background (19.3% vs 8.3%, *p* < 0.001) and be from a remote (3.7% vs 2.2%, *p* < 0.001) or very remote area (9.7% vs 1.5%, *p* < 0.001) (Table 1). The median time to relocation was 1.6 [IQR, 0.7–3.4] years (Table 2). Time from RRT commencement to relocation is shorter in patients from an ATSI background (*p* < 0.001), with a comorbidity (1.5 vs 2.1 years, *p* < 0.001) and who received facility HD compared to home therapies and transplant (1.4 vs 1.5 vs 3.9 years, respectively, *p* < 0.001). Time to relocation is also shorter as remoteness of residence changed from major city to very remote areas (2.0 vs 0.6 years, p for trend < 0.001).

**Table 2:** Time from commencement of renal replacement to relocation in relocation cohort

	Time to relocation (years)
Relocation Group	1.6 [0.7, 3.4]
Gender	
Male	1.7 [0.7, 3.4]
Female	1.6 [0.7, 3.3]
Race	
Caucasian	1.9 [0.8, 3.6]
Asian	1.9 [0.9, 3.8]
ATSI	0.9 [0.4, 2.0]
MPI	1.7 [0.8, 2.9]
Other	1.8 [0.7, 3.2]
Comorbidity	
Yes	1.5 [0.7, 3.0]
No	2.1 [0.9, 4.0]
Cause of Primary Renal Disease	
Glomerulonephritis	1.9 [0.8, 3.8]
Diabetes	1.4 [0.6, 2.9]
Hypertension	1.7 [0.7, 3.3]
Cystic disease	2.4 [1.0, 4.2]
Other	1.7 [0.8, 3.4]
RRT at relocation	
Facility HD	1.4 [0.6, 3.0]
Home Therapies	1.5 [0.7, 2.6]
Transplant	3.9 [2.3, 5.6]
Urbanity (from)	
Major City	2.0 [0.9, 3.8]
Inner Regional	1.8 [0.8, 3.3]
Outer Regional	1.4 [0.7, 2.9]
Remote	1.3 [0.5, 2.5]
Very remote	0.6 [0.3, 1.2]
Urbanity (to)	
Major City	1.9 [0.8, 3.6]
Inner Regional	1.8 [0.8, 3.5]
Outer Regional	1.1 [0.4, 2.4]
Remote	0.8 [0.4, 1.6]
Very remote	1.3 [0.7, 2.6]

Data expressed as median [intraquartile range]. Analysis by Log rank test for study cohort

All variables statistically significant (*P* < 0.05)

### Incidence and pattern of geographical relocation

Incidence of relocation in the total RRT cohort was 7.9 per 100 patient years (95% CI 7.4–8.5). Incidence increased across remoteness of residence within each geographical area reaching in major city 7.2 per 100 patient years (95% CI 6.6–7.8), in inner regional areas 5.9 per 100 patient years (95% CI 4.8–7.1), in outer regional areas 8.6 per 100 patient years (95% CI 6.9–10.3), in remote areas 13.5 per 100 patient years (95% CI 9.8–17.2) and in very remote areas 48.8 per 100 patient years (95% CI 45.6–51.9) (*p* < 0.001). Most relocations (4083, 70.6%) occurred within same remoteness of residence (Table 3) with more than half the relocations (56.5%) occurring within major cities (incidence 4.4 per 100 patient years). For within major city relocation only 7.4% were accompanied with a change of state indicating that more than 90% of major city to major city relocations occurred in the same city.

**Table 3:** Geographical relocation breakdown by direction of urbanity

	Relocation to					
Relocation from		Major City	Inner Regional	Outer Regional	Remote and Very Remote	Total
	Major City	3268 (89.9%)	267 (7.3%)	73 (2.0%)	26 (0.7%)	3634 (62.8%)
	Inner Regional	255 (31.9%)	464 (58.0%)	69 (8.6%)	12 (1.5%)	800 (13.8%)
	Outer Regional	129 (21.9%)	125 (21.2%)	306 (51.9%)	27 (4.6%)	590 (10.2)
	Remote and Very Remote	121 (15.9.0%)	32 (4.2%)	314 (41.3%)	83 (10.9%)	759 (13.1%)
	Total	3773 (65.2%)	888 (15.4%)	762 (13.2%)	296 (5.1%)	5783

Relocation rate by RRT modality increased from 5.4 per 100 patient years (95% CI 4.9–5.7) for transplant, 7.3 per 100 patient years (95%CI 6.9–7.7) for home therapies to 9.4 per 100 patient (95% CI 9.1–9.7) years for facility HD (*p* < 0.001).

For all RRT patients the net relocation by remoteness of residence was a gain of 0.12 per 100 patient years for major cities, 0.12 per 100 patient years for inner regional, 0.24 per 100 patient years for outer regional and 0.13 per 100 patient years for remote. There was a net loss of 0.65 per 100 patient years for very remote regions.

### Factors associated with geographical relocation

**Fig. 1:**
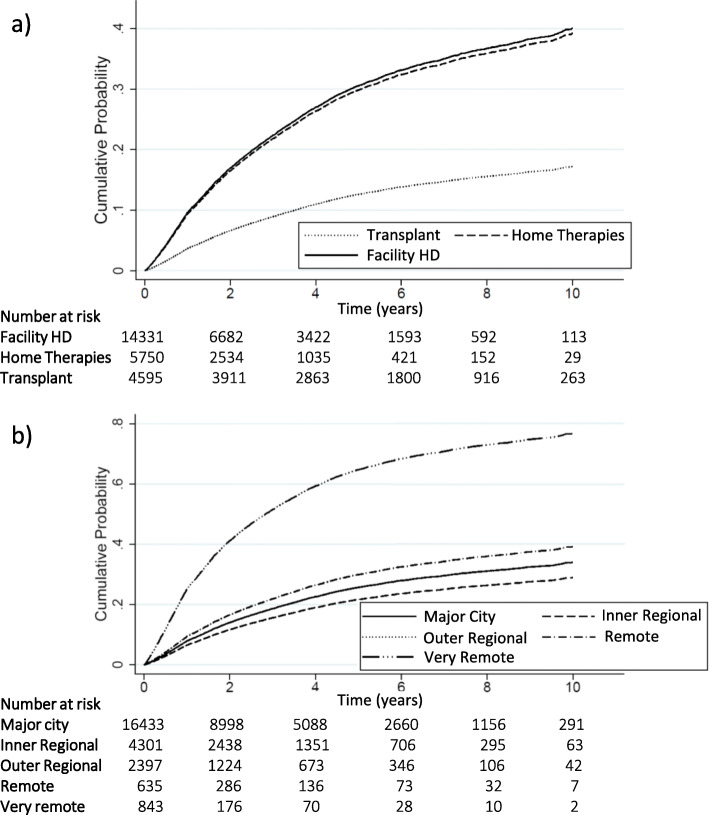
Cumulative incidence graph showing time to relocation by (**a**) renal replacement therapy modality and (**b**) remoteness of residence index, using mortality as a competing risk to calculate cumulative incidence function estimate. (Solid line for major city and dotted line for inner regional overlapping)

Variables associated with relocation was determined using Cox proportional hazard models (Table 4). A strong association was found for ethnicity, RRT at relocation and remoteness of residence. This remained the case in the competing risk analysis with transplant associated with a lower risk compared to facility HD as the RRT modality (SHR 0.37, 95%CI 0.34–0.40, *p* < 0.001) (Fig. 1). Ethnicity and remoteness of residence remained an independent risk for relocation with ATSI having a SHR of 1.18 (95%CI 1.06–1.31, *p* = 0.002), being from a remote region SHR 1.20 (95%CI 1.01–1.42, *p* = 0.034) and very remote region SHR 3.51 (95%CI 3.05–4.04, *p* < 0.001).

**Table 4:** Cox proportional hazard models and competing risk analysis, with death considered as a competing risk, examining risk factors for relocation

	Cox proportional hazards model Univariate analysis HR (95%CI)	*P*-value	Cox proportional hazards model Multivariate analysis aHR (95%CI)	*P*-value	Competing risk analysis Multivariate analysis SHR (95% CI)	*P*-value
Gender (M)	1.09 (1.03, 1.15)	0.001	–	–	–	–
Age (per decade)	0.87 (0.85, 0.88)	< 0.001	0.80 (0.78, 0.81)		0.75 (0.74, 0.77)	< 0.001
Race						
Caucasian	Reference	–	Reference	–	Reference	–
Asian	1.07 (0.97, 1.17)	0.14	0.98 (0.89, 1.08)	0.73	1.06 (0.96, 1.16)	0.27
ATSI	2.99 (2.80, 3.20)	< 0.001	1.21 (1.09, 1.34)	0.001	1.18 (1.06, 1.31)	0.002
MPI	1.60 (1.40, 1.83)	< 0.001	1.07 (0.97, 1.27)	0.19	1.14 (0.99, 1.31)	0.052
Other	1.46 (1.22, 1.75)	< 0.001	1.25 (1.04, 1.50)	0.01	1.27 (1.07, 1.51)	0.006
Comorbidity (Yes)	1.18 (1.12,1.24)	< 0.001	–	–	–	–
Cause of Primary Renal Disease						
Glomerulonephritis	Reference	< 0.001	–	–	–	–
Diabetes	1.27 (1.19, 1.37)	0.4	–	–	–	–
Hypertension	0.97 (0.88, 1.06)	< 0.001	–	–	–	–
Cystic disease	0.78 (0.70, 0.88)	0.1	–	–	–	–
Other	0.94 (0.87, 1.02)	–	–	–	–	–
RRT at relocation						
Facility HD	Reference	–	Reference	–	Reference	–
Home Therapies	1.08 (1.02, 1.15)	0. 01	1.02 (0.96, 1.09)	0.54	0.97 (0.91, 1.04)	0.40
Transplant	0.37 (0.34, 0.39)	< 0.001	0.29 (0.27, 0.32)	< 0.001	0.37 (0.34, 0.40)	< 0.001
Urbanity (from)						
Major City	Reference	–	Reference	–	Reference	–
Inner Regional	0.83 (0.77, 0.89)	< 0.001	0.83 (0.77, 0.90)	< 0.001	0.82 (0.76, 0.89)	< 0.001
Outer Regional	1.19 (1.01, 1.30)	< 0.001	1.03 (0.93, 1.13)	0.59	0.99 (0.91, 1.09)	0.98
Remote	1.86 (1.62, 2.13)	< 0.001	1.18 (1.01, 1.38)	0.04	1.20 (1.01, 1.42)	0.034
Very remote	6.46 (6.46, 7.06)	< 0.001	3.61 (3.20, 4.10)	< 0.001	3.51 (3.05, 4.04)	< 0.001

*HR* Hazard ration, *aHR* adjusted hazard ratio, SHR: subdistribution hazard ratio

## Discussion

This is the first study to our knowledge describing geographical relocation in RRT patients, or any patient group with chronic diseases, on a national level. We found 23.9% of Australian RRT patients between 2005 and 2015 relocated, with a relocation incidence of 7.9 per 100 patient years. The proportion is higher than previous estimates of 5%, found in New South Wales in Australia [17], and 15%, calculated from the difference in age adjusted dialysis prevalence for urban vs rural areas in a Japanese prefecture [4]. The increased proportion in our analysis likely resulted from including relocation within same remoteness of residence index and examining a national registry over a 10-year period.

We highlighted the high relocation rate with increasing remoteness of residence index, with patients relocating more frequently and earlier from outer regional, remote and very remote regions towards the major city and inner regional areas. There are likely two main factors encouraging patients on RRT to geographically relocate; distance to a treatment centre and rurality. Distance to a treatment centre with corresponding travel time may add hours to an already lengthy treatment session. The higher incidence of relocation found in facility HD compared to transplant is evidence of the burden frequent travel for treatment could impose.

There is a clear decrease in prevalence of facility HD with increased distance and travel time from a treatment centre [3–6]. Prevalence is estimated to decrease by 5.5% for every 10 min increase in travel time between residence and treatment centre [4] with prevalence significantly decreasing once travel time exceeds 15 min [5, 6]. An increase in the distance between patient residence and treatment centre is also associated with an increase in all-cause mortality [2, 18] and mortality from infection [18]. It was previously hypothesized that the decrease in prevalence with increasing travel time is a result of increased mortality or withdrawals [2, 19], however our analysis supports the notion that patient relocation is also an important factor.

The second factor encouraging geographical relocation is rurality. Australian rural patients with chronic diseases undergo fewer diagnostic or therapeutic interventions compared to urban centres, [10, 19–23] highlighting an inequality in the provision of health services. This extends to rural RRT patients who have a lower health service utilisation, physician visits and proportion of dialysis care [4, 5, 12, 17]. Australian RRT patients in regional districts, compared to major cities, have a lower survival and higher risk of hospitalisation [12, 17] while transplant patients in major cities are less likely to have an acute rejection in the first 6 months post-transplant [12]. While we found the absolute number of relocations from rural areas to be low when compared to urban centres it does highlight the greater burden which RRT patients experience in these regions.

We found the risk of relocation among indigenous population significant. Indigenous populations on RRT remain susceptible to relocation given rural urbanity in Canada [13] and Australia where relocation rates can reach 50% in some communities [24]. The burden of illness suffered by native populations is further complicated by the difficulty in providing specialised tertiary level care to geographically isolated small communities.

Geographic relocation for better access to specialised treatment facilities does come with costs in the form of significant financial, cultural and psychological pressure on the patient [15]. Patients may face family separation and a lack of social support [13, 14] with significant detrimental effects on quality of life and ability and willingness to maintain treatment regimens [15]. Some patients struggle with the idea of relocating by themselves and are faced with the burden of relocating the whole family [14]. A better understanding of the reasons behind relocation (personal or as a necessity for easier access to treatment), factors influencing this decision and the impact it causes is needed. Such information would also inform health policy makers to enable optimization of health resource allocation and development of efficient treatment networks. This is particularly true in the Australia where provision of coordinated comprehensive care for RRT patients can be a challenge in the setting of a large landmass and low population density with a sizable rural population.

Our study has several limitations. The data entered in the ANZDATA registry is submitted by each treatment centre, and the registry does not verify the accuracy of these reports or missing information. The dependency on postcode change to define a relocation likely underestimated the magnitude of our results. Since ANZDATA registry identified relocations at the end of the calendar year, the actual time to relocation is likely shorter. Our study was an observational analysis hence we could not prove direction of causality. We were also unable to include patients who relocated prior to commencing RRT or patients who were managed conservatively for ESRD. Finally, besides distance to treating centre, other psychosocial factors may influence the decision for relocation including patient and family supports, housing instability, financial security, and need for home care which we were unable to explore. Future studies are required to explore these factors.

## Conclusion

We determined the relocation incidence and prevalence in RRT in Australia. We also established factors associated with higher relocation rates. Further studies could be undertaken to determine the cause for relocation and the effect relocation imposes on quality of life and mortality.

## Data Availability

The datasets generated and/or analysed are available from the Australia and New Zealand Dialysis and Transplant Registry (ANZDATA) https://www.anzdata.org.au/. Data is available according to the ANZDATA release guidelines.

## Abbreviations

ANZDATA: Australia and New Zealand Dialysis and Transplant Registry
ATSI: Aboriginal and Torres Strait Islander
HD: Hemodialysis
HR: Hazard ratio
MPI: Maori Pacific Islander
RRT: Renal replacement therapy
SHR: Sub-hazard ratio
